# Does the presence of a pharmacist in primary care clinics improve diabetes medication adherence?

**DOI:** 10.1186/1472-6963-12-391

**Published:** 2012-11-13

**Authors:** Beverly Mielke Kocarnik, Chuan-Fen Liu, Edwin S Wong, Mark Perkins, Matthew L Maciejewski, Elizabeth M Yano, David H Au, John D Piette, Chris L Bryson

**Affiliations:** 1 Division of General Internal Medicine, University of Washington, 329 Ninth Ave, Campus Box 359780, Seattle, WA, 98104, USA; 2 Northwest Center for Outcomes Research in Older Adults, VA Puget Sound Health Care System, 1100 Olive Way, Suite 1400, Seattle, WA, 98101, USA; 3 Department of Health Services, University of Washington, Box 357660, Seattle, WA, 98195, USA; 4 Center for Health Services Research in Primary Care, Durham VA Medical Center, 508 Fulton St, Durham, NC, 27705, USA; 5 Division of General Internal Medicine, Duke University Medical Center, 2301 Erwin Rd, Durham, NC, 27710, USA; 6 Center for the Study of Healthcare Provider Behavior, Los Angeles VA 16111 Plummer St (152), Los Angeles, CA, 91343, USA; 7 Department of Health Services, University of California, 650 Charles Young Dr. S. 31-269 CHS, Box 951772, Los Angeles, CA, 90095, USA; 8 Department of Medicine, University of Washington, RR-512 Health Sciences, Box 356420, Seattle, WA, 98195, USA; 9 VA Center for Clinical Management Research, VA Ann Arbor Healthcare System, 2215 Fuller Rd., Mail Stop 152, Ann Arbor, MI, 48105, USA; 10 Department of Internal Medicine, University of Michigan School of Medicine, 1500 E. Medical Center Drive, Ann Arbor, MI, 48109, USA

**Keywords:** Pharmacist, Medication adherence, Diabetes mellitus, Oral hypoglycemic agent, Patient-centered medical home

## Abstract

**Background:**

Although oral hypoglycemic agents (OHAs) are an essential element of therapy for the management of type 2 diabetes, OHA adherence is often suboptimal. Pharmacists are increasingly being integrated into primary care as part of the move towards a patient-centered medical home and may have a positive influence on medication use. We examined whether the presence of pharmacists in primary care clinics was associated with higher OHA adherence.

**Methods:**

This retrospective cohort study analyzed 280,603 diabetes patients in 196 primary care clinics within the Veterans Affairs healthcare system. Pharmacists presence, number of pharmacist full-time equivalents (FTEs), and the degree to which pharmacy services are perceived as a bottleneck in each clinic were obtained from the 2007 VA Clinical Practice Organizational Survey—Primary Care Director Module. Patient-level adherence to OHAs using medication possession ratios (MPRs) were constructed using refill data from administrative pharmacy databases after adjusting for patient characteristics. Clinic-level OHA adherence was measured as the proportion of patients with MPR >= 80%. We analyzed associations between pharmacy measures and clinic-level adherence using linear regression.

**Results:**

We found no significant association between pharmacist presence and clinic-level OHA adherence. However, adherence was lower in clinics where pharmacy services were perceived as a bottleneck.

**Conclusions:**

Pharmacist presence, regardless of the amount of FTE, was not associated with OHA medication adherence in primary care clinics. The exact role of pharmacists in clinics needs closer examination in order to determine how to most effectively use these resources to improve patient-centered outcomes including medication adherence.

## Background

Oral hypoglycemic agents (OHA) are a therapeutic mainstay for type 2 diabetes patients with mild to moderate disease [1]. However, a systematic review of 20 studies indicated that OHA adherence rates range from 36-93% [2]. More recent studies have found that 65-78% of patients have an adherence rate of greater than 80% for oral hypoglycemic agents, which is a commonly accepted threshold for clinical effectiveness [1,3,4].

Tasks typically done by clinical pharmacists, including following a diabetes care algorithm [5], diabetes education, medication counseling, education about home glucose monitoring [6,7], adjustment of hypoglycemic regimens [6] and patient refill reminders, [8] have all been associated with improved medication adherence. A systematic review found that pharmacist-provided patient care was associated with reductions in hemoglobin A1c, LDL cholesterol, blood pressure and adverse drug events [9]. However, these studies usually evaluated specific pharmacy-based interventions rather than the influence of pharmacists in uncontrolled, “real-world” primary care practices.

Like many health systems nationally, the Veterans Health Administration (VA) is undergoing a major transformation of primary care to team-based care, by implementing a patient centered medical home (PCMH) model system wide [10,11] to improve access, coordination, and continuity of care. Pharmacists have been recommended as a standard component of patient-centered medical homes [12], but their impact on OHA adherence has not been studied. Pharmacists in VA primary care clinics may have a clinically oriented role by providing counseling and education to patients taking diabetes medications. However, pharmacists in VA may also be limited to a purely dispensing role or simultaneously manage both clinical and dispensing tasks. VA is a particularly good system for studying this question due to its nationally integrated computerized patient medical records, including pharmacy refill information, which provides an estimate for medication adherence. In VA, primary care is provided through clinics based at VA hospitals and their affiliated community based outpatient clinics [13]. A VA parent hospital and its affiliated community clinics share the management structure and support, such as administration, feedback regarding treatment quality, and centralized support for care management.

The purpose of this study was to examine whether the presence of pharmacists in VA primary care clinics was associated with improved adherence to OHAs among patients with type 2 diabetes. We hypothesized that the proportion of patients within a primary care clinic who would be adherent to OHAs was higher when a pharmacist was present, since these staff would be able to focus on barriers to obtaining refills and improvements in adherence counseling. We also examined the relationship between the degree of bottleneck in pharmacy services at the primary care clinics as reported by directors of primary care clinics and clinic-level medication adherence.

## Methods

### Data

This study used data from VA’s national administrative and pharmacy databases linked with a national survey of 196 primary care clinics focused on staffing and organizational barriers to care. Patient demographics, diagnoses, and utilization data from Fiscal Years (FYs) 2005–2007 were obtained from the VA Vital Status File, and medication adherence measures were constructed using pharmacy records from the FY2007 Decision Support System (DSS) National Outpatient Pharmacy Extracts. All data were measured prior to VA’s implementation of PCMH in April 2010.

To assess whether the integration of pharmacy staff in primary care clinics, as indicated by the PCMH model, improves adherence to OHAs, we obtained data on pharmacist presence and the number of full-time equivalent (FTE) pharmacists in a clinic from the 2007 Veterans Health Administration (VHA) Clinical Practice Organizational Survey—Primary Care Director Module [14]. The survey was sent to 250 VA primary care clinics, which included 153 hospital-based clinics and 97 community-based satellite clinics. The overall survey response rate was 93% [14,15]. Our study sample included 196 clinics that provided complete responses to the questions about pharmacists and pharmacy services. Human Subjects approval was obtained from Seattle, Durham and Ann Arbor VA Medical Centers.

### Study sample

Patients with type 2 diabetes were identified using a previously validated algorithm based on two or more outpatient or one inpatient diabetes related ICD-9 diagnosis in FY2005 or FY2006 [16]. Users of OHAs, including sulfonylureas, metformin, and thiazolidinediones, were defined as those who had at least two non-partial (>30 day) fills of the same OHA in FY2006. Selection of these three OHAs was driven by the VA formulary and constituted over 98% of all OHA prescriptions in VA. For inclusion in our study, patients were required to be non-institutionalized, prescribed at least one class of OHA, and be alive through the end of FY2007. Patients were excluded if they were prescribed any insulin except neutral protamine Hagedorn (NPH) to remove those who may have transitioned to insulin therapy during the observation period. We did not exclude patients on NPH insulin because it was frequently used as an adjunct to oral therapy. Further exclusion criteria included: patients with a nursing home or extended hospital stay in FY2007, patients seen in VA clinics outside the continental United States, or patients treated at VA contracted clinics. If patients were seen at multiple VA primary care clinics, they were assigned to the one with the most visits or, if the numbers of visits were equal, were randomly assigned to one of the clinics. The final study sample included 280,603 diabetes patients who received care from 196 primary clinics for which data were also available from the primary care survey. These patients were a subset of a sample documented in a prior study [17].

### Adjusted clinic-level medication adherence

Adherence to OHAs was estimated at the patient level using ReComp, a validated medication-possession ratio (MPR) algorithm described previously [18,19]. MPR has been shown to be correlated with hemoglobin A1c values in patients with diabetes [20]. Adherence was measured for each class of OHA in 90-day increments for all of FY2006 and FY2007 and the adherence values for the first quarter of FY2007 were used as the dependent variable. The 90-day time period was chosen since refill adherence for this 90-day duration is associated with physiologic outcomes [18]. Patients were considered adherent if they had medication available for 80 percent or more of the period, i.e., MPR>=80%. If patients were taking more than one OHA, the proportion of days covered during the period for each medication class was averaged into an overall adherence value. For example, a patient on both metformin and a sulfonylurea who had three 30-day fills (100%) of the sulfonylurea with one 30-day fills (33%) of metformin would have an average adherence score of 66.5%, which would be considered nonadherent to the overall regimen. Patients in VA obtain medication refills by either phoning the pharmacy, using an online patient portal (My Health*e*Vet), using automated calling systems, or making a clinic appointment.

Clinic-level adherence measures were then calculated based on the proportion of OHA adherent patients within a clinic. For example, if 140 of 200 patients in a clinic had 80% or greater adherence, then the clinic would have an adherence rate of 70%. To account for differences in patient characteristics across clinics that might influence clinic-level adherence scores, we risk adjusted clinic-level adherence scores using a hierarchical logistic regression model (see Wong, Piette et al. [17] for more details). Specifically, the model first estimated adherence at the patient level adjusting for patient characteristics known to impact adherence such as race and the presence of comorbid depression. Random intercepts were also included for parent hospitals and primary care clinics affiliated (i.e., nested) with each parent hospital to account for other facility characteristics that might influence adherence, such as the presence of automated phone systems for prescription refills. Predicted clinic-level adherence was then obtained from the patient-level model using the mean characteristics for a given clinic.

### Measuring the role of pharmacists in the clinic

We used four measures, including presence of pharmacists, two pharmacist FTEs measures, and perception of pharmacy services as a bottleneck in primary care clinics, derived from the VHA Clinical Practice Organizational Survey.

Questions pertaining to the role of the pharmacist were completed by the primary care director at each clinic. In the survey, respondents were asked:with “pharmacists” among the listed options. If pharmacists were indicated, respondents were asked:

"“Which of the following types of clinicians or other staff are included as members of your primary care program?”"

"“What is the combined total of whole and part full-time equivalent employees (FTEs) currently allocated to your primary care program for this type of clinician?”"

For the current study, we measured the availability of pharmacists in each clinic in three ways: 1) a binary variable indicating whether any pharmacists were noted in the survey as included in the primary care program; 2) pharmacist FTE per 10,000 primary care patients to account for clinic size; and 3) pharmacist FTE per 100,000 primary care patient encounters to account for clinic workload.

For the perception of pharmacy services, respondents were asked: with “Pharmacy services” among the list of services. Questions regarding bottlenecks in primary clinics were adapted from clinic manager surveys administered by the Minimizing Error, Maximizing Outcome Study [21]. In their study, questions measuring the degree of bottleneck in different areas of a primary care clinic were asked after physicians pointed out process factors affecting quality and errors during focus groups [21,22]. These factors included inadequate resources, ambiguous or nonexistent policies, poor communication and errors in information systems [23]. We constructed a categorical measure of the pharmacy as a perceived bottleneck using the response options “little/no extent,” “some extent,” “moderate extent”, and “large extent”. We included all clinics because patients receiving primary care at clinics with no pharmacist have alternative means of obtaining medications from VA. However, none of these measures could delineate the exact role of pharmacists in primary care clinics (i.e. anticoagulation management, medication titration for chronic diseases such as hypertension and diabetes, patient medication counseling, facilitation of refills, patient consultation, etc.).

"“To what extent are each of the following a patient flow ‘bottleneck’ for your primary care clinic?”"

### Statistical analysis

The level of analysis was the clinic (n=196). We performed ordinary least squares (OLS) regression with adjusted clinic-level medication adherence, i.e., proportion of OHA adherent patient in a clinic, as the outcome variable and pharmacist availability as the exposure of interest. Four separate analyses were performed, one for each of four different measures of pharmacist exposure described above.

Several auxiliary analyses were performed to evaluate model robustness and sensitivity to characteristics of the sample. Community-based clinics were significantly more likely to report having pharmacists than hospital-based clinics, therefore we performed sensitivity analysis of the effect of pharmacist availability separately within those two clinic-type subgroups. Results were similar, so we report the pooled sample results below. We also performed a sensitivity analysis using a 1-year adherence measure with similar results using a 90-day adherence measure, therefore we presented the results of 90-day adherence in this paper. We performed the Breusch-Pagan test [24] of heteroskedasticity to determine if clinic size affected the error variance, and found no evidence of heteroskedasticity. Also, a post-hoc power analysis by simulation [25] was performed to determine our statistical power to discern an effect of pharmacist presence in OLS models. Results indicated that with a sample of 196 clinics, assuming an alpha level of 0.05 (two-tailed) and a 1% effect size for clinic-level adherence, the estimated power was greater than 99%. For an assumed effect size of 0.5%, the estimated power was 95.5%. All analyses were performed using SAS Version 9.2 (SAS Corporation, Cary, NC USA).

## Results

### Descriptive statistics of adherence and pharmacist resources in primary care clinics

Among the 196 clinics, the number of patients with diabetes ranged from 241 to 7,399. Community based clinics on average had fewer diabetes patients than hospital-based clinics (1,019 versus 1,641, p<0.001). The average adjusted clinic-level adherence to OHAs was 70.7% and ranged from 57.5% to 79.1%. The average adjusted clinic-level adherence for primary care clinics with and without a pharmacist was 70.4% and 71.3%, respectively. Pharmacists were present in 70.4% of primary care clinics, with no significant difference in pharmacist availability between hospital-based clinics and community-based clinics (67.7% versus 75.8%, p=0.245). For those clinics that reported having a pharmacist, the average pharmacist FTE was 3.13 and ranged from 0.2 to 13 (Standard Deviation (SD)=2.43).

### Descriptive statistics of patients in primary care clinics

Patients were predominantly older and male (Table 1). The average number of primary care visits per year was 3.9. The two most common comorbidities were hypertension (64.1%) and coronary artery disease (19.1%). Patients seen in clinics with a pharmacist differed from those seen in clinics without a pharmacist across a number of characteristics including race and incidence of chronic obstructive pulmonary disease and post-traumatic stress disorder.

**Table 1:** Descriptive statistics of study sample

**Measure**	**Overall**	**Pharmacist in clinic**	
		**YES**	**NO**
**Clinic Characteristics**			
# Clinics	196	138	58
Community-based Clinic (%)	33.7	36.2	27.6
Study subjects per facility (mean/SD)	1,432 (870)	1,457 (891)	1,370 (822)
Minimum	241	241	253
Maximum	7,399	7,399	4,262
Total patients per facility (mean/SD)	28,162 (18,973)	28,873 (19,397)	26,469 (17,972)
Minimum	4,925	4,925	5,694
Maximum	108,694	108,694	90,024
Pharmacist FTE/10,000 clinic patients	1.03 (1.27)	1.46 (1.23)	N/A
Pharmacist FTE/100K patient encounters	1.63 (2.13)	2.31 (2.21)	N/A
**Patient Characteristics**			
# Subjects	280,603	201,127	79,476
Age (mean/SD)	67.4 (11.0)	67.3 (11.0)	67.5 (10.9)
Female (%)	2.3	2.3	2.2
Married (%)	63.9	63.1	65.9
White Race (%)	73.0	71.5	76.8
Free care due to disability (%)	36.4	36.1	37.1
Free care due to low income (%)	35.3	35.8	34.2
No free care (%)	28.3	28.1	28.7
DCSI Score (mean/SD)	3.8 (1.2)	3.8 (1.2)	3.8 (1.2)
Primary care visits (mean/SD)	3.9 (3.0)	3.9 (3.1)	3.7 (2.8)
Total visits (mean/SD)	7.7 (9.7)	7.8 (10.0)	7.3 (8.8)
DCG Risk score (mean/SD)	0.9 (0.6)	0.9 (0.6)	0.9 (0.6)
Cumulative in hospital days (mean/SD)	0.2 (2.0)	0.2 (2.0)	0.2 (1.9)
Hospitalized during FY06 Q4 (%)	2.8	2.8	2.9
Hypertension (%)	64.1	64.8	62.1
Peripheral vascular disease (%)	1.3	1.3	1.3
Ischemic heart disease (%)	19.1	19.1	19.2
Myocardial Infarction (%)	1.3	1.3	1.4
Stroke (%)	2.5	2.6	2.1
Other cerebrovascular disease (%)	1.0	1.1	1.0
Chronic obstructive pulmonary disease (%)	7.6	7.4	8.1
Congestive heart failure (%)	4.0	3.9	4.3
Chronic renal failure (%)	2.6	2.7	2.3
Atrial fibrillation or flutter (%)	6.1	6.1	6.0
Dementia (%)	1.0	1.0	0.9
Alcohol abuse (%)	2.1	2.0	2.1
Drug Abuse (%)	7.1	7.0	7.5
Post-traumatic stress disorder (%)	7.2	7.4	6.8
Depression (%)	8.8	8.8	8.7
Schizophrenia (%)	1.7	1.8	1.6
Other mental illness (%)	1.7	1.8	1.6
Warfarin (%)	6.2	6.1	6.4
NPH insulin usage (%)	9.0	9.7	7.3

SD = Standard deviation; FTE = Full time equivalents; DCSI = Diabetes complications severity index; DCG = Diagnostic cost group; NPH = Neutral protamine Hagedorn; FY06 = Fiscal year; Q4 = Fourth quarter.

### Regression results

**Figure 1:**
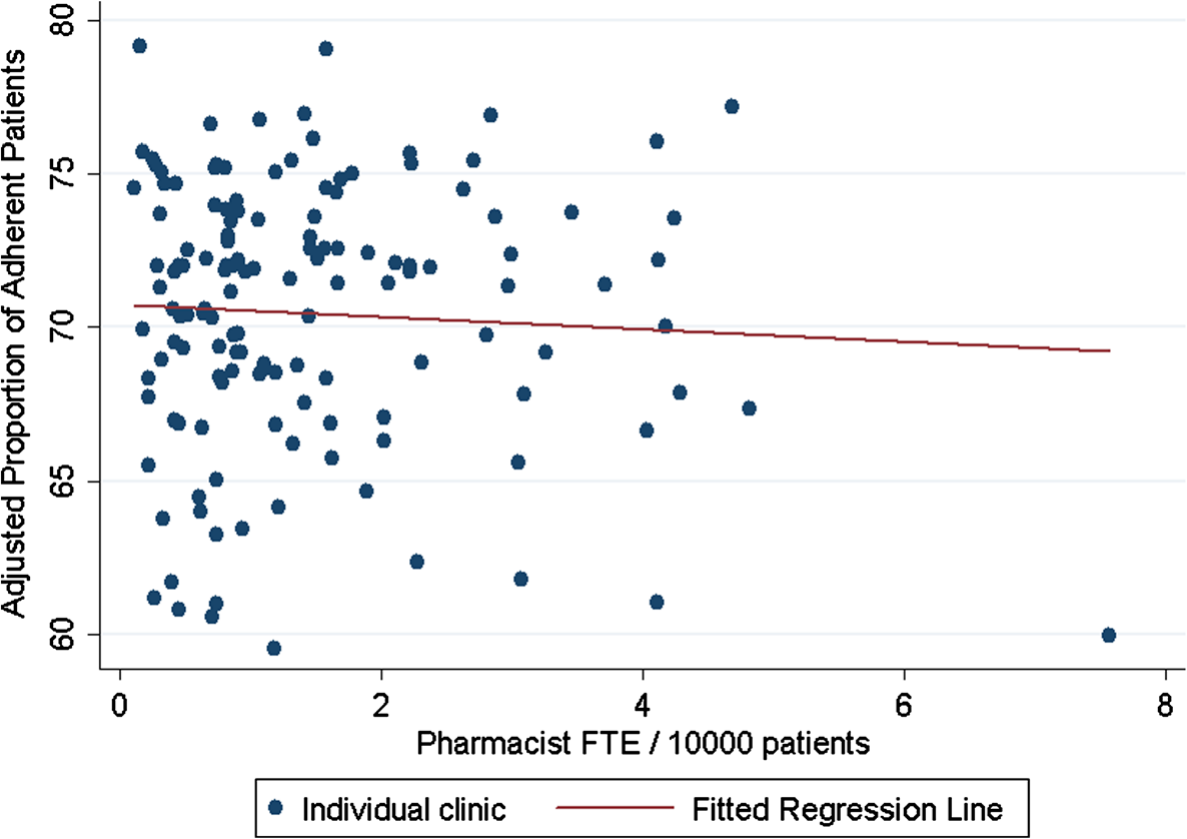
Association between pharmacist full-time equivalents (FTE) per 10,000 primary care clinic patients and the proportion of patients adherent in primary care clinics.

**Figure 2:**
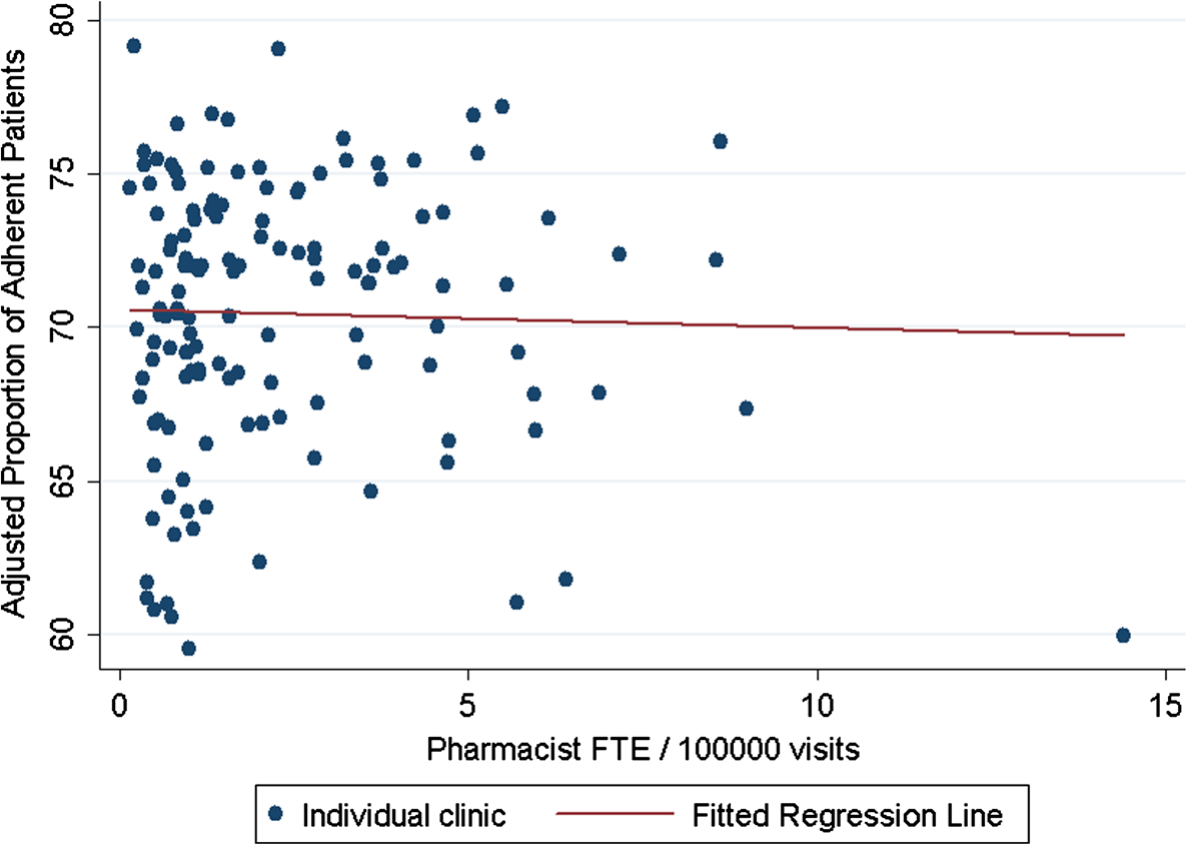
Association between pharmacist full-time equivalents (FTE) per 100,000 primary care clinic patient visits and the proportion of patients adherent in primary care clinics.

The model testing the effect on adherence of any pharmacist presence showed no statistically significant relationship (p=0.227). When adjusting for clinic size, pharmacist FTE per 10,000 primary care patients (p=0.494) and pharmacist FTE per 100,000 primary care encounters (p=0.723) were also not associated with clinic-level adherence (Table 2, Figures 1 and 2). The amount of variation in clinic-level adherence explained by pharmacist presence or pharmacist FTE was small with R-squared values ranging from 0.0009 to 0.0074. All results were similar when examining community-based and hospital-based clinics separately.

**Table 2:** Associations between clinic-level medication adherence and pharmacist presence and pharmacy is a bottleneck

	**# Facilities**	**Coefficient**	**Pr > Chi-Sq**	**R-Squared**
**Pharmacist in clinic**				
Pharmacist in clinic (0,1)	196	−0.0083	0.2269	0.0074
Pharmacist FTE per 10K patients, when FTE>0	138	−0.0021	0.4938	0.0034
Pharmacist FTE per 100K encounters, when FTE>0	138	−0.0006	0.7228	0.0009
**Pharmacy is bottleneck (reference=no bottleneck)**				
Some extent	196	−0.0138	0.0570	
Moderate extent	196	−0.0194	0.0310	0.0421
Large extent	196	−0.0296	0.0102	

### Pharmacy bottleneck

**Figure 3:**
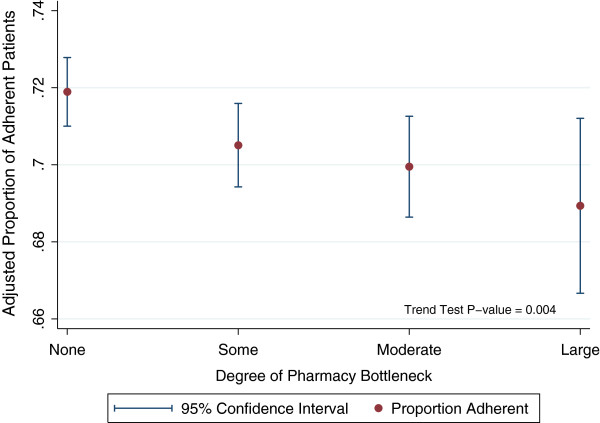
Association between the perception of pharmacy services as a bottleneck and the proportion of patients adherent in primary care clinics.

Of the 196 clinics in our sample, those where pharmacy services were perceived as a bottleneck had poorer adherence (Wald statistic for trend, p=0.004). Adherence scores for clinics with a high degree of bottleneck (p=0.010), a moderate degree of bottleneck (p=0.031) and a small degree of bottleneck (p=0.057) were lower compared to clinics with little or no bottleneck (Table 2, Figure 3). The amount of variation in clinic-level adherence explained by pharmacy bottleneck measures was 4.2%.

## Discussion

This study assessed the association between the presence of pharmacists and the proportion of diabetes patients adherent to OHAs within VA primary care clinics. The results indicate that adherence scores are not significantly better within clinics that have a pharmacist present, regardless of the amount of FTE pharmacist staff available. However, the perception of pharmacy services as a bottleneck was associated with lower clinic-level medication adherence. While the percent of variation in clinic-level adherence explained by each pharmacy measure was generally small (< 5%), relative to pharmacist presence measures, the perception of the pharmacy as a bottleneck had substantially greater explanatory power.

Our clinic-level findings generally differ from prior patient-level studies demonstrating the value of having pharmacists present in primary care clinics [9,26-29]. Most often, these studies evaluated a specific function or task related to the pharmacist as a mechanism for improving diabetes care. In contrast, the current study evaluated pharmacist presence and therefore characterizes their impact on adherence in more “real world” settings.

As the structure of clinics changes towards a multi-disciplinary team approach using the PCMH, there will be an increasing component of healthcare provided by the non-physician care management team [30]. The PCMH calls for pharmacists to be part of this team and pharmacists have been included in prior PCMH demonstration projects [31]. The goals of including pharmacists in the PCMH are to optimize medication regimens, improve management of chronic diseases, and lower barriers to adherence through assisting in refilling and renewal of medications [12,32]. VA is working towards complete implementation of a PCMH within all primary care clinics and has routinely included pharmacists in primary care clinics since 2010 [33]. Our results suggest that without a clearly defined role, simply including pharmacists in clinics may not improve adherence to OHAs.

As in other healthcare systems, several organizational factors in VA that are beyond pharmacists influence may contribute to adherence problems. For example, barriers in refilling medications could potentially mitigate the benefits of pharmacists. These barriers include trouble getting through on phone lines [34], lower health related internet use among veterans living in rural locations [35], and difficulty getting to clinics [36,37]. Although the role of a pharmacist in the PCMH model should ideally include helping to decrease barriers to accessing or refilling medications, pharmacists may currently have much different job functions. For example, if pharmacists are staffing anti-coagulation clinics, spending time dispensing medications, or completing other such tasks, then they will not have the ability to engage in activities such as medication education and counseling which have been shown to positively impact medication adherence for patients with diabetes.

The association between the degree of bottleneck in pharmacy services and clinic-level medication adherence suggests factors related to pharmacy services as perceived by the primary care director impair the timely dispensing of medications to patients. This result is consistent with a prior study showing pharmacy processes such as communication between inpatient discharge team, outpatient physicians and pharmacists were associated with a reduction in health care visits and readmission [38]. Although prior studies have shown the value of including pharmacists on general medicine teams [36,39], our results suggest that the mere presence of a pharmacist is not enough to improve medication adherence. Instead, organization resources, policies and procedures must be in place in order for pharmacists to effectively perform job duties.

Our study has several strengths. Data were drawn from a large, national administrative data system that includes information on several important confounding patient-level variables. The power for our study was excellent, with a greater than 99% ability to detect a 1% difference in clinic-level adherence between clinics with and without a pharmacist. Also, we were able to link administrative data to a nationwide clinic-level survey of pharmacist availability that is rarely available in other health systems and had a 93% response rate from 250 clinic representatives.

Several limitations exist in our study. First, we are unable to distinguish pharmacists based on variation across clinics in their training, duties, or activities. If pharmacists have more clinically-oriented roles at some locations, but are limited to only dispensing medications at other locations, then our findings are biased towards the null hypothesis, masking potential pharmacist benefits in facilities that have a more clearly defined adherence-support role for their pharmacists. Future research should also examine the exact roles of pharmacists within VA and whether there is any association with medication adherence based on specific clinical functions. Second, the measures of pharmacist availability and FTEs used in the study are reported by primary care directors, but have not been validated. However, these measures are the best that are currently available because administrative data do not indicate whether pharmacists are specifically assigned to a primary care clinic. Third, the number of community-based clinics reporting the presence of a pharmacist was higher than we expected, and some clinics may have reported on pharmacists at parent VA facilities who may have little day to day involvement in that community clinic. However, the results remain the same when we conducted the analysis including hospital-based clinics only, so we cannot attribute the observed null findings exclusively to reporting on off-site pharmacists. Finally, this study was conducted prior to the implementation of PCMH in VA. Further studies should examine the functions of pharmacists in that new primary care model.

## Conclusions

In summary, the study shows that the proportion of patients adherent to OHAs in VA primary care clinics was not associated with the availability of pharmacists in that clinic. However, pharmacy services perceived as bottlenecked are associated with lower adherence. Our results call for further research disentangling the role of pharmacists in VA primary clinics and an examination of how these roles can most efficiently affect patient outcomes such as improved medication adherence.

## Competing interests

DHA reports serving as a consultant for Bosch Inc. and receiving grants from Gilead Sciences. All other authors declare that they have no competing interests.

## Authors’ contributions

BMK and CLB conceived the study. BMK, CFL, ESW, MP and CLB participated in its design. MP prepared the data for analysis and performed the statistical analyses. BMK drafted the manuscript. All authors participated in the analysis and interpretation of data and the critical revision of the manuscript for important intellectual content. CFL and CLB obtained funding for this project. All authors have read and approve the final manuscript.

## Pre-publication history

The pre-publication history for this paper can be accessed here:

http://www.biomedcentral.com/1472-6963/12/391/prepub
